# Selective translational repression of HIV-1 RNA by Sam68DeltaC occurs by altering PABP1 binding to unspliced viral RNA

**DOI:** 10.1186/1742-4690-5-97

**Published:** 2008-10-28

**Authors:** Kim Marsh, Vanessa Soros, Alan Cochrane

**Affiliations:** 1 Department of Molecular Genetics, University of Toronto, Toronto, Ontario, Canada; 2 Sea Lane Biotechnologies, 1455 Adams Drive, Menlo Park, California 94025, USA

## Abstract

HIV-1 structural proteins are translated from incompletely spliced 9 kb and 4 kb mRNAs, which are transported to the cytoplasm by Crm1. It has been assumed that once in the cytoplasm, translation of incompletely spliced HIV-1 mRNAs occurs in the same manner as host mRNAs. Previous analyses have demonstrated that Sam68 and a mutant thereof, Sam68ΔC, have dramatic effects on HIV gene expression, strongly enhancing and inhibiting viral structural protein synthesis, respectively. While investigating the inhibition of incompletely spliced HIV-1 mRNAs by Sam68ΔC, we determined that the effect was independent of the perinuclear bundling of the viral RNA. Inhibition was dependent upon the nuclear export pathway used, as translation of viral RNA exported via the Tap/CTE export pathway was not blocked by Sam68ΔC. We demonstrate that inhibition of HIV expression by Sam68ΔC is correlated with a loss of PABP1 binding with no attendant change in polyadenosine tail length of the affected RNAs. The capacity of Sam68ΔC to selectively inhibit translation of HIV-1 RNAs exported by Crm1 suggests that it is able to recognize unique characteristics of these viral RNPs, a property that could lead to new therapeutic approaches to controlling HIV-1 replication.

## Introduction

Expression of the full coding potential of the HIV-1 genome is dependent upon a number of post-transcriptional processes. The primary 9 kb transcript from the integrated provirus can be spliced into over 30 mRNAs through suboptimal splicing events [1-4]. Resulting HIV-1 mRNAs can be grouped into three classes: the unspliced, 9 kb class, encoding Gag and GagPol; the singly spliced, 4 kb class, encoding Vif, Vpr, Vpu and Env; and the multiply spliced, 2 kb class, encoding Tat, Rev and Nef. Incompletely spliced mRNAs are normally retained in the nucleus but the virus has evolved a mechanism for the transport of the 9 kb and 4 kb viral mRNAs to the cytoplasm. The Rev protein is translated in the cytoplasm, then shuttles into the nucleus where it multimerizes on the Rev Response Element (RRE) contained in the introns of the incompletely spliced HIV-1 mRNAs. Once Rev binds to the RNA, its nuclear export signal (NES) interacts with Crm1 and mediates export to the cytoplasm [5,6].

HIV-1 gene expression may be controlled at several steps including transcription, splicing, polyadenylation, nuclear export and translation [3,4,7]. All of these processes depend upon host cell factors [8]. Recent work in our laboratory has focused on Sam68, a member of the STAR/GSG family of proteins [9]. These proteins contain an RNA binding motif, the KH domain, embedded within a larger conserved GSG (Gld1, Sam68, GRP33) domain, which mediates multimerization. Sam68 is a nuclear, non-shuttling protein, and contains both proline- and tyrosine-rich domains mediating binding to multiple kinases (i.e. Src, Lck, Sik/BRK, ZAP-70) through SH3 and SH2 domains, respectively [9,10]. Given its interaction with kinases involved in signal transduction, Sam68 has been suggested to serve as a signal mediator that affects multiple cellular processes including cell cycle regulation, tumour suppression, alternative splicing, and RNA 3' end formation [9-17]. More pertinent to HIV-1, overexpression of Sam68 and other members of the GSG family have been shown to significantly enhance HIV-1 gene expression [18-21]. Sam68 can also enhance expression of HIV-1 mRNAs exported to the cytoplasm via the constitutive transport element (CTE) of Mason-Pfizer monkey virus by promoting utilization by the translational apparatus of the cell [22]. Two groups have reported that depletion of Sam68 results in the loss of HIV-1 structural protein expression in several cell lines [23-25].

In contrast to the full length protein, a truncation mutant of Sam68 lacking the C-terminal 112 amino acids, Sam68ΔC, is a potent inhibitor of HIV-1 protein expression [19,21]. Unlike Sam68, Sam68ΔC is localized predominantly in the cytoplasm and its inhibitory function requires this distribution [21]. Therefore, differences in activity between Sam68 and Sam68ΔC likely reflects the different protein-protein interactions available in the different compartments of the cell. Previous experiments in our lab showed that Sam68ΔC induced accumulation of HIV-1 4 kb mRNAs into perinuclear bundles suggesting that it might act by sequestering the viral RNA from the translational apparatus [21]. In this study, we set out to define the mechanism and specificity of Sam68ΔC inhibition. We show that Sam68ΔC specifically inhibits RRE containing mRNAs. We also demonstrate that depolymerization of microfilaments disrupted the perinuclear bundles, dispersing the viral RNA throughout the cytoplasm, but failed to restore the synthesis of the HIV-1 structural proteins (Gag, Env). This finding suggests that the block to expression is at the level of engagement with the translational apparatus. Subsequent analysis of HIV-1 *env *mRNA distribution in polysome gradients in the presence and absence of Sam68ΔC supports this conclusion. Our studies determined that Sam68ΔC has no effect on viral RNA polyadenylation or poly(A) tail length. Inhibition of translation by Sam68ΔC was not associated with any changes in viral RNA localization, abundance, or processing but is correlated with changes in the composition of the mRNP. We show that Sam68ΔC inhibition of HIV-1 mRNA translation is accompanied by a reduction in PABP1 association with the affected mRNAs.

## Results

### Susceptibility to Sam68ΔC repression is conferred by the nuclear export pathway

**Figure 1:**
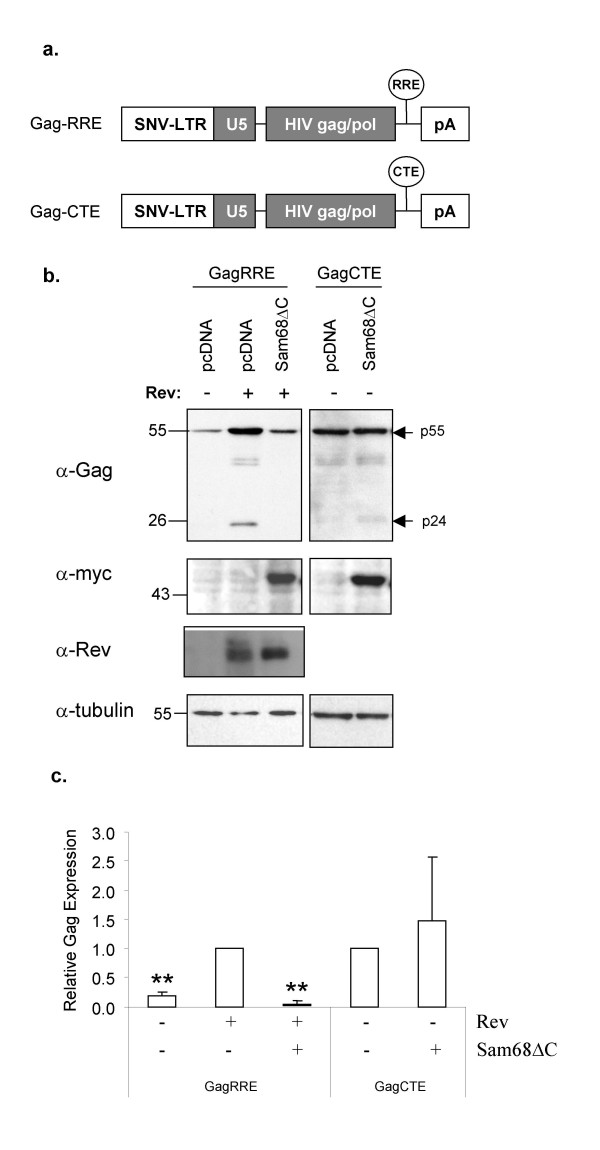
**Sam68ΔC selectively suppresses expression of mRNAs exported by the Rev/RRE complex**. a) An illustration of the two HIV-1 Gag expression constructs used: Gag-RRE and Gag-CTE. b) 293T cells were transfected with either Gag-RRE or Gag-CTE plasmids in the absence (-) or presence (+) of expression vectors for Rev and Sam68ΔC. Forty-eight hours post-transfection, cell lysates were prepared, fractionated on SDS-PAGE gels and blotted. Blots were probed to detect levels of Gag (p55 and p24, α-Gag), Sam68ΔC (α-myc), Rev (α-Rev) and tubulin (α-tubulin). c) Quantitation of Gag expression over multiple assays, results being normalized to tubulin levels. Asterisks denote samples determined to have significantly different levels of p24 expression from that seen with Gag-RRE and Rev at a p < 0.01.

The ability of Sam68ΔC to selectively suppress expression of the 9 kb and 4 kb classes of HIV-1 mRNAs suggested that there is some unique feature that renders them susceptible to repression. Cellular mRNAs use the Tap export pathway, while HIV-1 9 and 4 kb RNAs are incompletely spliced and contain sequences preventing their export by Tap [26-33]. These incompletely spliced HIV-1 RNAs are exported from the nucleus via the interaction of HIV-1 Rev with the host protein Crm1 [5,6,34]. Two possible explanations for repression of the 9 kb and 4 kb HIV-1 RNAs by Sam68ΔC are immediately apparent: either they contain unique RNA sequences recognized by Sam68ΔC or export via the Crm1 pathway marks the viral RNA for inhibition. To address this question directly, we examined the ability of Sam68ΔC to inhibit expression of HIV-1 Gag RNAs utilizing different nuclear export elements; the constitutive transport element (CTE) from Mason-Pfizer Monkey virus that interacts directly with Tap (Gag-CTE), or the RRE that requires Rev and Crm1 (Gag-RRE) (Fig. 1a) [35]. Gag RNA generates a 55 kDa polyprotein that is subsequently processed by the viral protease into matrix (p17), capsid (p24), nucleocapsid (p9) and p6. Gag expression was measured by anti-Gag western blot in which the p55 precursor and p24 are detected. GagRRE expression is dependent upon Rev and is reduced to baseline by Sam68ΔC (Fig. 1b,c). Parallel western blots demonstrated that Sam68ΔC does not markedly alter Rev levels (Fig. 1b). In contrast to the Gag-RRE reporter, Sam68ΔC had no significant effect on expression from Gag-CTE (Fig. 1b,c). This demonstrates that it is not the Gag sequence, but rather the RRE and/or the Crm1 export pathway that dictates inhibition by Sam68ΔC.

### Perinuclear bundling of HIV-1 RNA by Sam68ΔC does not account for translation inhibition

**Figure 2:**
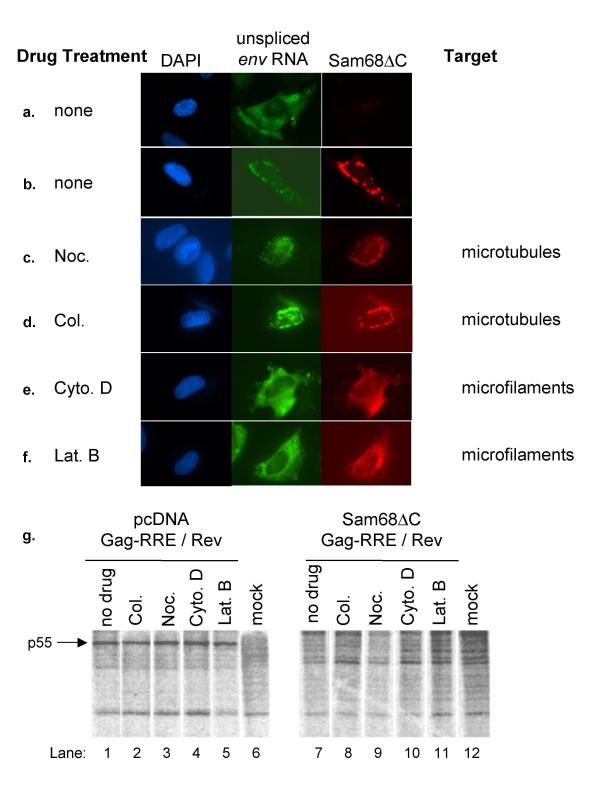
**Sam68ΔC maintains translational repression of viral mRNAs when perinuclear bundles are dissolved**. To analyze the requirement of the cytoskeleton for the formation of Sam68ΔC-induced perinuclear bundles, the effect of disrupting microtubules or microfilaments on viral RNA subcellular distribution was examined. As a comparison, HeLa cells were co-transfected with Rev, the HIV-1 *env *expression plasmid pgTat and pcDNA (a) or Sam68ΔC (b) and untreated prior to fixation (none). In parallel, Hela cells transfected with pgTat, Rev and Sam68ΔC were treated 48 h post-transfection with nocodazole (Noc.) (c), colcemid (Col.) (d) to disrupt microtubules or cytochalasin D (cyto. D) (e) or latrunculin B (Lat. B) (f) to depolymerise microfilaments for 2 hours prior to fixation. Locations of the unspliced pgTat (env) RNA and Sam68ΔC were determined by *in situ *hybridization and immunofluorescence respectively. Nuclei were stained with DAPI (g) To determine the impact of altered HIV-1 RNA subcellular distribution on its translation, HeLa cells were co-transfected with Rev, Gag-RRE and pcDNA or Sam68ΔC. 48 hours post-transfection, cells were treated with the indicated drug for 2 hours to depolymerise either the microtubules or microfilaments. Then ^35^S-methionine was added to the media and incubated for 4 hours prior to harvest. Cell lysates were prepared and incubated with anti-Gag antibody. Immunoprecipitates were run out on 10% SDS-PAGE and exposed to a phosphor screen. The position of the Gag p55 band is indicated.

Previously, we reported that Sam68ΔC expression induced accumulation of both unspliced viral RNA and Sam68ΔC in bundles at the outer periphery of the nucleus [21]. As shown in Fig. 2a, in the absence of Sam68ΔC, HIV-1 *env *RNA is distributed throughout the cytoplasm. The formation of the perinuclear bundles upon co-expression of Sam68ΔC, as seen in Fig. 2b, suggested that Sam68ΔC might be sequestering the RNA from the translational apparatus. If true, disruption of these complexes should restore translation of the viral RNAs. We examined the effect of various agents which disrupt the cytoskeleton on the integrity of the Sam68ΔC/viral RNA perinuclear bundles, and the expression of viral proteins (Fig. 2c–f)). To minimize secondary drug effects, the minimum amount of each drug required to depolymerize its target in one hour was used (data not shown). While treatment of cells with either nocodazole or colcemid to disrupt microtubules had no effect on localization of viral RNA or Sam68ΔC (Fig. 2c,d), disassembly of the microfilaments by treatment with cytochalasin D or latrunculin B resulted in dispersal of both viral RNA and Sam68ΔC throughout the cytoplasm (Fig. 2e, f). The distribution of the viral RNA which has been released from the perinuclear bundles is similar to that seen in the absence of Sam68ΔC (Fig. 2a).

Next we questioned whether the released viral RNA was translated. Cells were transfected with Gag-RRE and Rev expression vectors in the presence or absence of Sam68ΔC. Forty-eight hours post-transfection, cells were treated with the drugs as before and synthesis of viral protein was monitored by incubation in the presence of ^35^S methionine. Cells were lysed, the ^35^S-labelled Gag immunoprecipitated, the immunoprecipitates run out on SDS-PAGE gels, and the gels exposed to a phosphorscreen overnight (Fig. 2g). The 55 kDa Gag polyprotein (p55) was not detected in the immunoprecipitates from the mock transfected cells but only in immunoprecipitates from cells transfected with Gag-RRE and Rev (Fig. 2g, lanes 1–5 versus 6). Therefore, the immunoprecipitation was specific and the p55 signal could be used as a measure of Gag-RRE translation. None of the drugs had any effect on the level of Gag expression in the absence of Sam68ΔC indicating that they had no significant effect on translation (Fig. 2g, lanes 1–6). None of the drugs induced expression of Gag in the presence of Sam68ΔC (Fig. 2g, lanes 7–12) despite latrunculin B/cytochalasin D shifting the subcellular distribution of both Sam68ΔC and the viral RNA. Therefore, integrity of the Sam68ΔC/viral RNA perinuclear bundles is not essential for translational repression. This observation suggests that Sam68ΔC inhibits viral RNA translation, not by changing its subcellular distribution, but blocking interaction with the translational apparatus. This effect could be achieved either through alterations in viral RNA structure or composition of the mRNP.

### Sam68ΔC inhibits HIV *env *RNA recruitment into heavy polysomes

**Figure 3:**
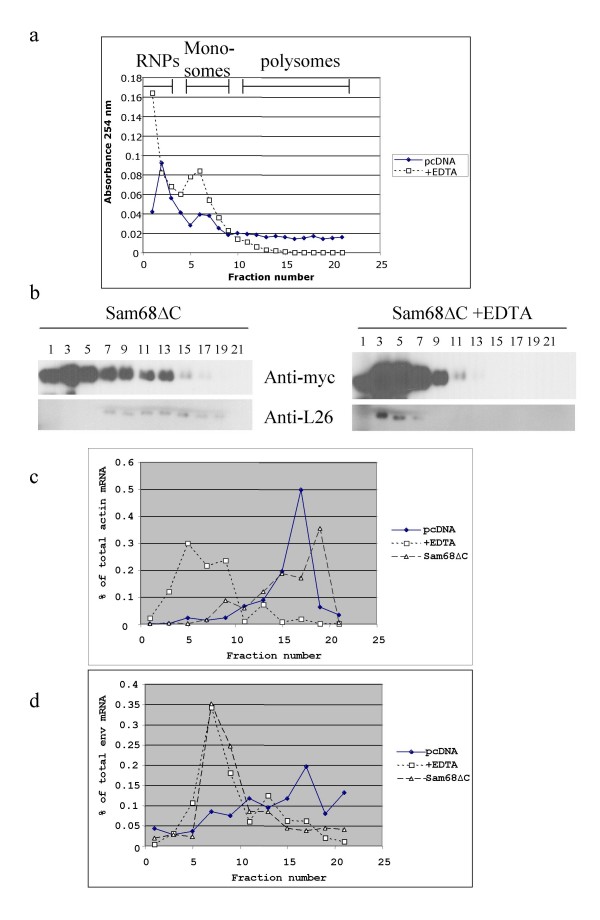
**Effect of Sam68ΔC on association of *env *RNA with polysomes**. Cells were co- transfected with Rev, the HIV-1 *env *expression plasmid pgTat and pcDNA or Sam68ΔC. 48 hours post-transfection cells were harvested and cytoplasmic extracts layered onto 15–50% linear sucrose gradients. Following centrifugation, gradients were fractionated and analyzed by monitoring (a) absorbance at 254 nm (b) distribution of Sam68ΔC and ribosomal protein L26 by western blotting or (c) actin and (d) unspliced *env *RNA distribution by QRT-PCR. To ascertain whether profiles were dependent upon the integrity of polysomes, an aliquot of the cell lysate was treated with EDTA (+EDTA) to dissociate ribosomal subunits prior to fractionation on the gradients.

To confirm that Sam68ΔC was acting at the level of translation, cytoplasmic extracts were fractionated on linear sucrose gradients (Fig. 3). Fractions collected were subsequently analyzed for the presence of Sam68ΔC, ribosomal protein L26, actin mRNA and unspliced HIV *env *RNA. As shown in Fig. 3b, Sam68ΔC is predominately found at the top of the gradient but a significant amount is present in the heavier fractions consistent with previous observations of an association between Sam68 and ribosomes [36]. Addition of EDTA to disrupt polysomes resulted in a shift in Sam68ΔC distribution to the top of the gradient, suggesting that there might be interaction of Sam68ΔC with polysomes. Parallel analysis of actin mRNA (Fig. 3c) revealed that the bulk of this RNA is found within heavy polysomes in the presence or absence of Sam68ΔC. EDTA addition induced a shift in distribution to lighter gradient fractions consistent with an association with polysomes. In contrast, unspliced HIV-1 *env *RNA underwent a shift in distribution from being predominately in the polysome fraction to predominately in the mRNP/monosome fraction in the presence of Sam68ΔC (Fig. 3d). The distribution of unspliced *env *RNA in the presence of Sam68ΔC significantly overlaps with that seen upon addition of EDTA consistent with a selective inhibition of translation of this mRNA.

### Mapping of domains within Sam68ΔC essential for repression of HIV-1 gene expression

**Figure 4:**
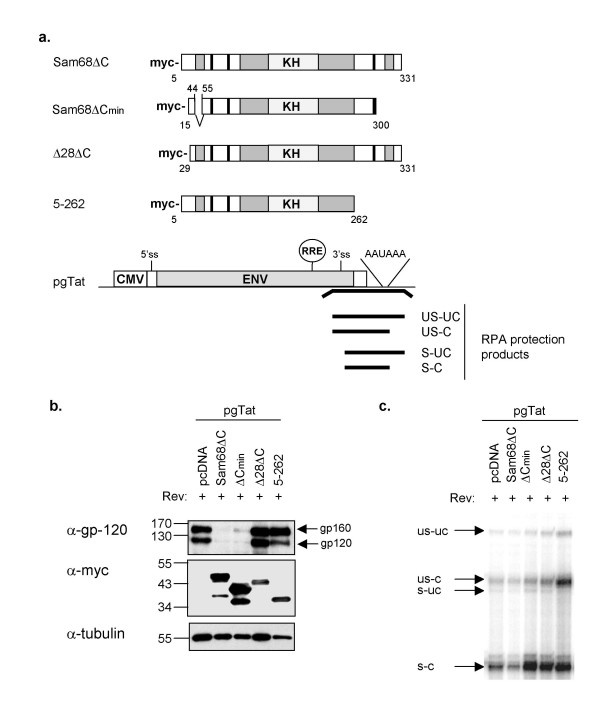
**Domain requirements for Sam68ΔC inhibition of gp120 protein synthesis from pgTat**. a) Illustration of Sam68ΔC domain structure, and various mutants thereof. Vertical black bars represent the proline rich domains; hatched boxes represent the RGG boxes; the horizontal bars represent the GSG domain; and the speckled box represents the KH domain. At the bottom is the illustration of the unspliced pgTat reporter construct, which encodes gp120. The black bar below pgTat indicates the position of the RPA probe used and the protection products expected. b) 293T cells were transfected with pgTat, Rev and the indicated Sam68ΔC mutants. Forty-eight hours post-transfection, cells were harvested and RNA and protein extracted for analysis. A representative western blot indicating Env (gp120) expression in the presence of Sam68ΔC and various mutants thereof is shown. Blots were reprobed with anti-myc to confirm protein expression and anti-tubulin to normalize for loading. c) RNA analysis of pgTat by RNase protection assay. The four protection products are indicated: unspliced-uncleaved (us-uc), unspliced-cleaved (us-c), spliced-uncleaved (s-uc) and spliced-cleaved (s-c). gp120 is translated from the us-c isoform of the RNA. 20 μg of total RNA was input to the assay.

Sam68 contains a number of well-defined domains that mediate specific RNA binding (KH-domain), non-specific RNA binding (RGG-boxes) or protein-protein interactions (proline-rich domains and tyrosine-phosphorylation sites) [9]. We made a number of deletions of Sam68ΔC to define a minimal inhibitory mutant (Fig. 4a,b). As shown by western blots (Fig. 4b), all mutants were equally expressed and subsequent immunofluorescence microscopy confirmed that all were localized to the cytoplasm (data not shown). Previous analyses have determined that the region spanning amino acids (a.a) 269–321 is essential for the inhibitory property of Sam68ΔC [37]. In our work, Sam68ΔCmin, spanning amino acids 14 to 300 with an internal deletion of amino acids 45 to 54 (encompassing an RGG box), was the minimal construct that retained significant inhibitory activity (Fig. 4b). In contrast, deletion of the first 28 (Sam68Δ28ΔC) or last 70 (Sam68:5–262) amino acids of Sam68ΔC resulted in a loss of inhibitory activity (Fig. 4b). These observations indicate that domains at the N- and C-terminus of Sam68ΔC are essential to its inhibitory activity.

Splicing, cleavage, and polyadenylation are tightly coupled events within the nucleus. However, in the case of HIV-1 RNA, numerous forms of viral RNA are generated, some of which have failed to undergo one or more of these steps. This is essential for the production of the HIV-1 structural proteins and therefore, the HIV-1 lifecycle. The HIV-1 *env *reporter, pgTat, expresses gp160/120 from the unspliced, cleaved mRNA (see Fig. 4a) [11]. Previously, we showed that full-length Sam68 increases the amount of unspliced, cleaved pgTat mRNA available for polyadenylation, export and translation into gp160/120 [11]. We have also shown that restoring nuclear accumulation of Sam68ΔC by addition of a heterologous nuclear localization signal (NLS) converted the protein into a stimulator of Rev function comparable to full length Sam68 [21]. In light of these results, we wanted to assess whether Sam68ΔC inhibition is due to alterations in the abundance or processing of *env *RNA.

We examined the extent of splicing and cleavage of pgTat RNA by RNase protection assay (RPA). As illustrated in Fig. 4a, the RPA probe used in this analysis spans both the 3' splice site and the polyadenylation site, yielding four RNase protection products: unspliced, uncleaved (US-UC); unspliced, cleaved (US-C); spliced, uncleaved (S-UC); and spliced, cleaved (S-C) (Fig. 4a). Sam68ΔC did not cause any marked change in the amount of US-C RNA that could account for the loss of gp160/120 expression but a reduction in levels of S-C RNA was consistently observed (Fig. 4c) that might reflect effects on either viral RNA splicing or S-C RNA stability. In contrast, the mutant, Sam68:5–262, increased the abundance of US-C RNA (Fig. 4c). The stimulation of cleavage of unspliced RNA by Sam68:5–262 is consistent with the known activity of full length Sam68 in promoting RNA polyadenylation previously reported by our laboratory [11] and define that different domains of Sam68 are required for inhibitory versus stimulatory activity.

### Inhibition by Sam68ΔC is not associated with changes in polyadenylation of *env *RNAs

**Figure 5:**
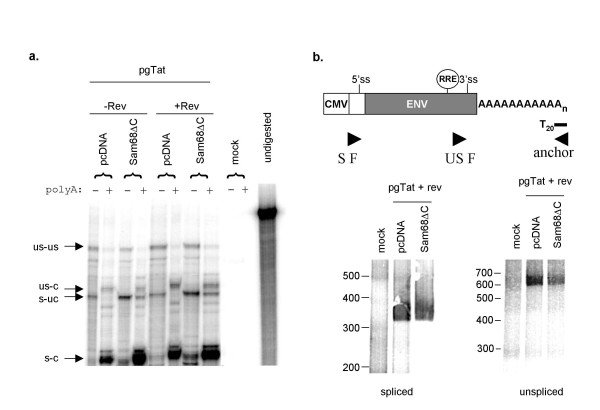
**Sam68ΔC does not alter the polyadenylation status of the affected viral RNAs**. Cells were transfected with pgTat with (+Rev) or without (-Rev) Rev in the absence (pcDNA) or presence of the various Sam68ΔC mutants. Total RNA was harvested and used in the assays below. a) 20 μg of total RNA was selected using oligo(dT) beads and both the polyA- and polyA+ fractions were input into the RNase protection assay. b) RACE-PAT analysis. The illustration at top shows the position of the anchor primer used to make cDNA and the relative positions of the PCR primers. The anchor primer can anneal anywhere along the length of the polyA tail in order to make cDNA, and the resultant PCR generates a smear representing various lengths of polyA tail. Amplicons to either spliced or unspliced RNAs were generated using S F or US F primers, respectively, and the anchor primer. Products were analyzed by fractionation on PAGE gels, and sizes of products determined by comparison to markers.

De-adenylation of mRNA is a common form of translational repression [38]. To assess changes in the polyadenylation state of *env *mRNAs in the presence of Sam68ΔC, total RNA was extracted and the polyadenylated RNA isolated using oligo(dT)_25 _beads. Given that cleavage and polyadenylation are tightly coupled processes, all cleaved RNAs are expected to have a poly(A) tail and bind to the oligo(dT)_25 _column. Appearance of cleaved RNA in the poly(A)- fraction would be indicative of a de-adenylation event. Analysis of the poly(A)- and poly(A)+ fractions by RPA revealed that the fractionation was successful; uncleaved *env *RNAs (US-UC and S-UC) being predominantly in the poly(A)- fraction and cleaved versions (US-C and S-C) in the poly(A)+ fraction (Fig. 5a). Sam68ΔC did not shift the distribution of the unspliced RNAs between the fractions, indicating that the US-C form of pgTat RNA retained a poly(A) tail of sufficient size to bind to the column (Fig. 5a).

To assay whether there were any significant changes in poly(A) tail length, we used a random amplification of cDNA ends, polyadenylation test (RACE-PAT) [39]. A primer consisting of a specific 15-mer sequence followed by 20 T residues was used to make cDNA. This primer anneals randomly along the length of the poly(A) tail, generating cDNAs with a range of lengths corresponding to where on the poly(A) tail cDNA synthesis was initiated. Using a reverse primer complementary to the 15-mer sequence and a *env *specific forward primer specific to either spliced (S F) or unspliced (US F) *env *RNA, we measured the length of the polyA tail of each RNA species (Fig. 5b). Without a poly(A) tail, the spliced amplicon is 340 nucleotides and the unspliced amplicon is 415 nucleotides. Therefore, the tail length of spliced pgTat RNA is approximately 10–60 nucleotides and that of the unspliced RNA is 200–250 nucleotides (Fig 5b). No change in amplicon size was observed upon addition of Sam68ΔC. Thus, the loss of gp120 expression upon Sam68ΔC co-transfection cannot be attributed to de-adenylation of unspliced *env *mRNA.

### Sam68ΔC alters the association of Rev-dependent viral RNAs with PABP1

**Figure 6:**
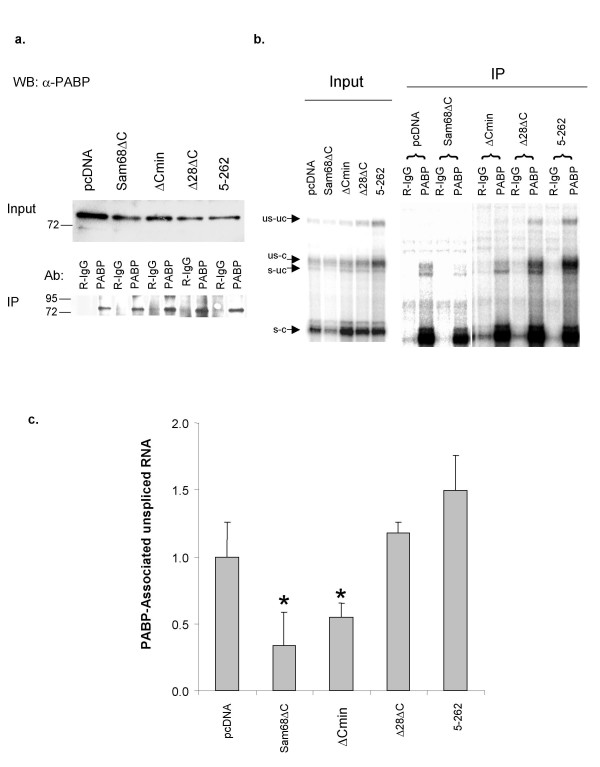
**Sam68ΔC inhibits the association of unspliced pgTat RNAs with PABP1**. 293T cells were transfected with pgTat and Rev in the absence (pcDNA) or presence of the various Sam68ΔC mutants. Cell lysates were prepared 48 h post-transfection and used in the assays below. a) PABP1 western blot showing the input and immunoprecipitated (IP) protein using either rabbit IgG (R-IgG) or anti-PABP1 (PABP). b) RNase protection assays showing input and immunoprecipitated (IP) pgTat RNAs. RNA was extracted from either rabbit IgG (R-IgG) or anti-PABP1 (PABP) precipitated samples and analyzed by RPA. c) Quantitation of RPA shown in (b). The ratio of US-C to S-C pgTat mRNA immunoprecipitated with anti-PABP1 was standardized to the input. The amount of US-C precipitated in the presence of pcDNA was set to 1.0. Error bars represent one standard deviation. Data was quantitated from 3 independent experiments. Asterisk indicates a value significantly different (p-value < 0.05) from samples transfected with pcDNA.

PABP1 has been shown to be an important promoter of mRNA translation through its direct interaction with eIF4G and resultant indirect interactions with the eIF4E cap binding protein [40,41]. Therefore, we examined PABP1 association with pgTat mRNA by RNP immunoprecipitation (RIP). PABP1 levels were not affected by expression of the Sam68 proteins and there was no change in epitope availability since precipitation of PABP1 was similar for each sample (Fig 6a). RNA analysis determined that pgTat RNAs were specifically precipitated with PABP1 as compared to control rabbit IgG RIPs (Fig. 6b). The S-C form of pgTat was the major isoform pulled down with anti-PABP-1 antibody but US-C RNA was also detected in the immunoprecipitates. Unexpectedly. some immunoprecipitation of both US-UC and S-UC was also detected particularly in the presence of the Sam68Δ28ΔC and Sam68:5–262. The reason for this is unclear but might reflect an indirect interaction of PABP1 with these RNAs (that lack a polyA tail). PABP1 association was quantitated by comparing the ratio of US-C to S-C pgTat RNA in the RIP compared to the input (Fig. 6c). Sam68Δ28ΔC and Sam68:5–262 had no significant effect on US-C RNA association with PABP1. Sam68ΔC and Sam68ΔCmin significantly reduced US-C RNA association with PABP1 (p < 0.05, Fig. 6b,c). Expression of Sam68ΔC also had no effect on the immunoprecipitation of RNPs containing the S/C form of pgTat RNA, consistent with Sam68ΔC being able to discriminate between the US-C and S-C forms of the viral RNA.

**Figure 7:**
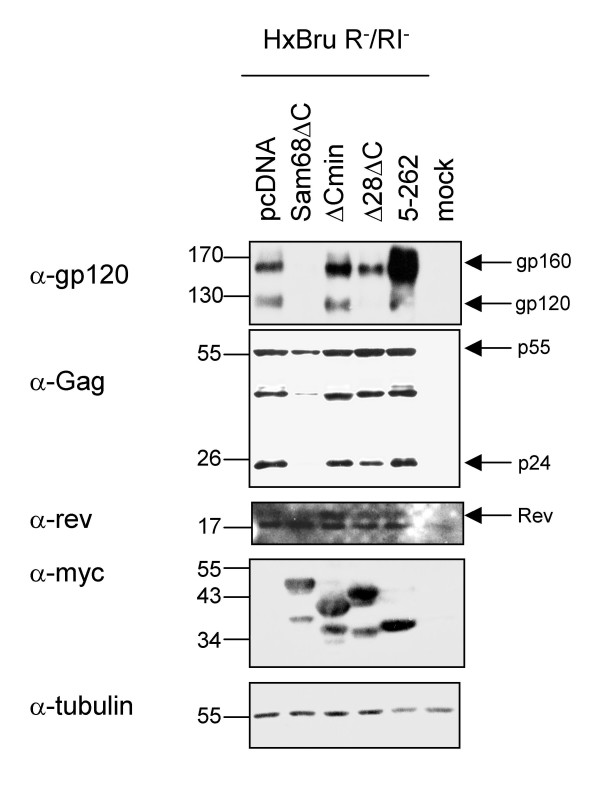
**Effect of Sam68ΔC mutants on protein expression from the HIV-1 provirus**. 293T cells were transfected with the proviral clone pHxBruR-/RI- in the absence (pcDNA) or presence of the various Sam68ΔC mutants. Western blots on total cell lysates harvested from 293T cells transfected with HxBruR^-^/RI^- ^and either pcDNA, Sam68ΔC or mutants thereof. p24 is expressed from the 9 kb, gp120 is expressed from the 4 kb, and Rev (top band) is expressed from the 2 kb class of proviral mRNA. Blots were reprobed with anti-myc to confirm protein expression and anti-tubulin to normalize for loading.

**Figure 8:**
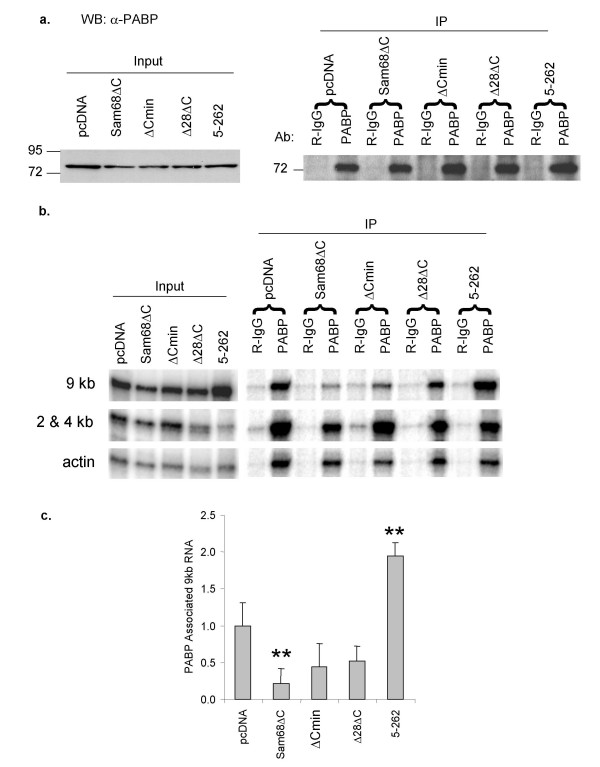
**Sam68ΔC inhibits the association of 9 kb proviral mRNAs with PABP1**. Cells were transfected with the proviral clone pHxBruR-/RI- in the absence (pcDNA) or presence of the various Sam68ΔC mutants. Cell lysates were prepared 48 h post-transfection and used in RIP assays. a) PABP1 western blot showing the input and immunoprecipitated (IP) protein using either rabbit IgG (R-IgG) or anti-PABP1 (PABP). b) RNase protection assays showing input and PABP1 immunoprecipitated (IP) HxBruR^-^/RI^- ^RNAs. RNA was extracted from either rabbit IgG (R-IgG) or anti-PABP1 (PABP) precipitated samples and analyzed by RPA. c) Quantitation of RPA shown in (b). The amount of 9 kb HxBruR^-^/RI^- ^mRNA relative to actin mRNA immunoprecipitated with anti-PABP1 compared to the input. The amount of 9 kb precipitated in the presence of pcDNA was set to 1.0. Error bars represent one standard deviation. Data was quantitated from 3 independent experiments. Asterisks indicate values significantly different (p-value < 0.01) from pcDNA.

To confirm that Sam68ΔC induced similar changes in PABP1 association in the context of the HIV-1 provirus, the experiment was repeated using a replication inactive form of the virus (HxBruR^-^/RI^-^). Sam68ΔC strongly reduced both p24 and gp160/120 expression from this construct (Fig. 7). However, the mutants have slightly different effects on the provirus than on the *env *reporter construct pgTat. While Sam68:5–262 enhances expression of gp160/120 and p24, Sam68Δ28ΔC and Sam68ΔCmin have little effect. None of the mutants had any effect on Rev or PABP1 protein levels (top band, Fig 7, Fig. 8a). PABP1 was immunoprecipitated uniformly in all samples (Fig. 8a). The proviral RNA associated with PABP1 was analyzed using an RPA probe that spans the 5' splice site within Gag, yielding 2 protection products. One protection product corresponds to the 9 kb and the other to the spliced forms (2 and 4 kb classes) of HIV-1 mRNAs. As translation of both the 9 kb (p24) and 4 kb (gp160/120) classes of viral RNAs are affected by Sam68ΔC, the band corresponding to the 2 and 4 kb classes could not be used for quantitation. Relative PABP1 association was quantitated by the ratio of 9 kb RNA to actin RNA in the RIP compared to the input. Sam68ΔC significantly decreased PABP1 association with HIV-1 9 kb RNA relative to pcDNA, while Sam68:5–262 significantly increased PABP1 association with HIV-1 9 kb RNA (p < 0.01, Fig. 8b,c). Both Sam68Δ28ΔC and Sam68ΔCmin showed slight reductions in PABP1 association with 9 kb viral RNA but these were not found to be significant even at a higher p-value (p < 0.05, Fig. 8b,c). Therefore, similar to the results seen with the pgTat vector, changes in viral protein expression are correlated with extent of interaction of the corresponding RNA with PABP1.

## Discussion

Recent studies have highlighted the changes in RNP composition that occur during mRNA processing, export and surveillance as the RNA moves from its site of synthesis through the nuclear pore to the translational apparatus. In particular, a number of hnRNP proteins are removed, the exon junction complex (EJC) is added following splicing then subsequently removed during translation, and there is an exchange of factors at the extreme 5' and 3' ends of the RNA (CBP20/80 and PABP2 are exchanged for eIF4E and PABP1, respectively) [42-44]. What remains unknown is the extent to which the protein composition of a specific RNP is determined by its sequence or its processing pathway. In the case of the EJC, the act of splicing deposits the complex ~20–25 nt 5' of the splice site in a sequence independent fashion [44]. In other instances, binding of factors to specific sequence elements (Zipcode localization elements, AU-rich elements) affects localization, stability or translation of an RNA [43]. It has generally been assumed that, despite an alternative export pathway, translation of incompletely spliced HIV-1 mRNAs occurs in the same manner as host mRNAs. However, in this study we provide evidence that Sam68ΔC is able to discriminate between RNAs based, in part, on the export pathway used (Fig. 1). What is unclear is whether export by Crm1 alone is sufficient to confer inhibition by Sam68ΔC or if additional features of the HIV-1 incompletely spliced RNAs (perhaps the Rev/RRE complex) provide additional recognition elements that confer regulation by this factor. Some data (not shown) supports the latter hypothesis.

Translational control has been studied extensively, and there are several well-defined mechanisms: changes in subcellular distribution (stress granules, P-bodies) [45,46], changes in RNA structure (de-adenylation, cytoplasmic adenylation)[38], destabilizing RNA elements (AREs) [47,48], and blocking eIF4E initiation (4E-BPs) [49] to name a few. Our data show that inhibition by Sam68ΔC is not dependent upon changes in mRNA subcellular distribution (Fig. 2). Polysome gradient analysis (Fig. 3) demonstrated that Sam68ΔC selectively blocks the association of unspliced HIV-1 *env *RNA with the polysome fraction, consistent with a block in one of the steps of translation. However, data acquired to date does not permit us to evaluate whether the block is at initiation or subsequent elongation. Future experiments will attempt to address this issue. These observations conflict with a previous report by Zhang et al that suggested the mechanism of inhibition was a block to nuclear export [37]. As evidence of this transport block, they performed nuclear/cytoplasmic fractionation in which Sam68ΔC sensitive mRNAs co-sedimented with the nuclear fraction. We clearly see that the inhibited mRNAs are in perinuclear bundles in the cytoplasm, in the presence of Sam68ΔC (Fig. 2b). However, their association with the microfilaments makes these mRNPs insoluble, and as a result they co-sediment with the nuclei and would be incorrectly scored as untransported (Fig. 2b, data not shown). Sam68ΔC does not affect target mRNA abundance (Fig. 4c), polyadenylation (Fig. 5a), or poly(A) tail length (Fig. 5b), only PABP1 binding (Fig. 6, 8). The reduction in PABP1 binding to Rev-responsive viral RNAs by Sam68ΔC could be achieved either by blocking PABP2 exchange for PABP1 following export, or removing PABP1 from the mRNA once it has engaged the translational apparatus. We believe that inhibition of PABP1 binding to an mRNA, by Sam68ΔC, represents a novel mode of translation inhibition but further studies will be necessary to identify the stage of cytoplasmic RNA processing being affected by Sam68ΔC.

Previous analyses had demonstrated that inhibition of HIV expression by Sam68ΔC was lost upon introduction of mutations that disrupted RNA binding capacity consistent with a direct interaction with the affected RNA being required [21]. Mutational analysis shown in this study indicates that additional domains of Sam68ΔC are essential to its inhibitory potential; amino acids 14 to 44 and amino acids 262 to 300. Since neither of these regions has been implicated in RNA binding, the findings suggest that these regions might be involved in mediating protein-protein interactions. Interestingly, the requirements for inhibition appear distinct from those promoting unspliced RNA polyadenylation and enhancement of HIV gene expression as further C-terminal mutations (Sam 5–262) converted the protein from an inhibitor to a stimulator. While the regions encompassed by Sam68ΔCmin were sufficient to inhibit HIV expression in several different assays, other data suggests that it might have reduced function. Sam68ΔC consistently inhibited expression of the incompletely spliced HIV-1 mRNAs and had a corresponding effect on PABP1 binding. However, the mutants had different effects on pgTat and HxBruR^-^/RI^- ^reporters. Importantly, the effect of the mutants on viral protein synthesis was mirrored in the effects they had on PABP1 binding. For instance, Sam68:5–262 had a limited effect on gp160/120 expression from pgTat, but increased the amount of gp160/120 and p24 from HxBruR^-^/RI^-^. Correspondingly, Sam68:5–262 had no significant effect on PABP1 binding to US-C pgTat mRNA but did significantly increase PABP1 binding to the 9 kb HxBruR^-^/RI^- ^mRNA. In contrast, Sam68ΔCmin was able to block expression of pgTat RNA but not the 9 kb and 4 kb RNAs of the provirus. Analysis of the effect of Sam68ΔCmin revealed that it decreased binding of PABP1 to US/C pgTat RNA but resulted in no or little alteration in PABP1 interaction with viral RNAs. Given that regions affecting inhibitory function are outside of the domain required for RNA binding (GSG and RGG motifs), the differences among the Sam68ΔC mutants likely reflect changes in the interaction with host factors. These factors could facilitate or inhibit remodeling of the viral RNP following export from the nucleus and their activity could vary depending upon subtle differences in the processing/composition of the RNPs being affected.

**Figure 9:**
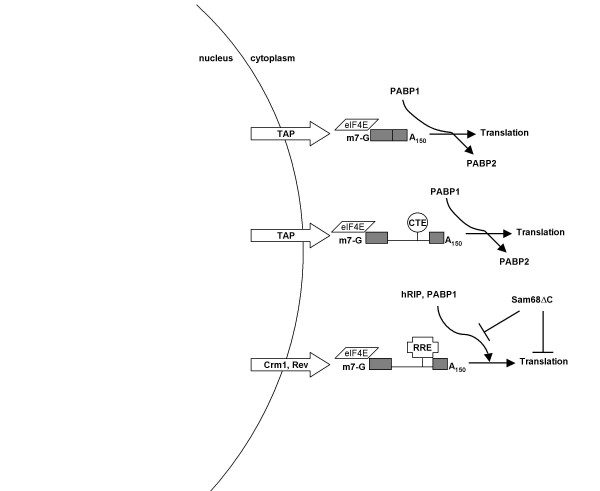
**Model of Sam68ΔC inhibition of HIV-1 protein expression**. The distinct processing pathways of fully spliced versus incompletely spliced viral RNAs result in differences in the composition of the RNPs that appear in the cytoplasm. Upon export, the RNPs undergo compositional changes including the exchange of PABP2 for PABP1 to become translationally active. Sam68ΔC interacts with unique components of the Crm1 exported RNPs to block the exchange of PABP2 for PABP1 or removes PABP1 from the affected RNA, resulting in a block to translation.

The perinuclear accumulation of viral RNA induced by Sam68ΔC, while not essential for the translational inhibition, is similar in phenotype to the effect seen upon depletion of human Rev-interacting protein (hRIP) [50]. This finding suggests that both Sam68ΔC and hRIP may be influencing a similar step in viral RNA metabolism. These observations are consistent with a model whereby the viral RNP and hRIP interact at the nuclear periphery (directly or indirectly) to initiate remodeling of the viral RNP to enhance its translation, a part of which is the exchange of PABP2 for PABP1. Sam68ΔC may interfere with this remodeling and prevent binding of PABP1, thereby inhibiting translation initiation (Figure 9). The recent identification of two RNA helicases (DDX1 and DDX3) that play essential roles in Rev function suggests that remodeling of the viral RNP plays an important role in ensuring efficient expression of the HIV-1 structural genes [51,52]. How and where these helicases act remains to be determined.

In summary, we have discovered that Sam68ΔC is able to use the features of Crm1 mRNA export to specifically inhibit translation of Rev-dependent HIV-1 mRNAs. Inhibition occurs by a novel mechanism: inhibiting the association of PABP1 with the target mRNA. Based on the data presented here, and other published work, we hypothesize that viral mRNAs transported via the Crm1 export pathway have a unique mechanism through which they engage the translation machinery, possibly involving hRIP, DDX1 and DDX3 (Fig. 9). If true, it would indicate that mRNAs using different export pathways have distinct RNP compositions and therefore, separate control mechanisms. Future research into the details of translation initiation for Rev-dependent HIV-1 mRNAs may lead us to discover further discrepancies from host mRNAs and lead to new therapeutic approaches to control HIV-1 gene expression.

## Materials and methods

### Expression constructs

The following constructs have been previously described: SV-Hygro, SV-H6Rev, CMVmyc3xterm [53], pDM128 [54], pgTat [55], Bl-env-HindIII [56], Sam68, and Sam68ΔC [21]. Gag-CTE and Gag-RRE plasmids were provided by Dr. K. Boris-Lawrie, Ohio State University. HxBruR^-^/RI^- ^was provided by Eric Cohen, Universite de Montreal. Sam68Δ28ΔC was generated by PCR using primers: 5'-CGGAATTCCCCTCGGTGCGTCTGA-3' and 5'-CGGGATCCAGTCACAGTGG-CACCTCT-3'. The amplicon was digested with EcoRI and BamHI and ligated into CMVmyc3xterm. Sam68:5–262 was made by EcoRI and EcoRV digest of Sam68ΔC and ligated into the EcoRI and SmaI sites of CMVmyc3xterm. Sam68ΔCmin was made in a stepwise fashion. First, Sam68:5–300 was generated by PCR using primers 5'-CCATTAACGCAAATGGGCGGTA-3' and 5'-CCGCTCGAGAACAGG-TGGAGG-3'. The amplicon was digested with EcoRI and XhoI and ligated into Sam68ΔC. To generate internal deletions, Quickchange mutagenesis (Stratagene) was carried out using Sam68:5–300 as a template: Δ14: 5'-GGGGAATTCGAGAAGATCGGGCCGCAGCTGGC-3' and 5'-GCAGCTGCGG-CCCGATCTTCTCGAATTCCC-3'; Δ(45–54): 5'-GCTTCCTCAC-CGGCCCGCTCGGGCCTCGCCC-3' and 5'-GGGCGAGGCCCGAGCGGGCCGG-TGAGGAAGC-3'. Bl-actin was generated by PCR from cDNA using primers: 5'-GCTACGAGCTGCCTGACG-3' and 5'-TCCTTCTGCATCCTGTCG-3'. The amplicon was cloned into the EcoRV site of Bluescript. Bl-SD-Gag was amplified from HxBruR^-^/RI^- ^using: 5'-CGCGGATCCGAAGTAGTGTGTGCCCGTCT-3' and 5'-CCCAAGCTTCCCTGCTTGCCCATACTATA-3'. The amplicon was digested with BamHI and HindIII and ligated into Bluescript.

### Cell lines and transfections

HeLa and 293T cells were maintained in Iscove's modified Dulbecco's medium (IMDM) supplemented with 10% fetal bovine serum (FBS), 50 μg/mL gentamycin sulfate and 2.5 μg/mL amphotericin B. Vectors were introduced to 293T cells by calcium phosphate transfection [57]. Vectors were introduced to HeLa cells by Fugene 6 transfection reagent (Roche) following manufacturer's protocol. Cells were harvested two days post-transfection.

### Antibodies

The following antibodies were used: mouse anti-myc (Invitrogen), mouse anti-tubulin (Sigma), mouse anti-gp120AK (courtesy of H. Schaal), mouse anti-p24 (clone 183-1112-5C), rabbit anti-L26 (Cell Signaling Technology) and rabbit anti-human PABP1 (aa 462–633) (courtesy of N. Sonenburg, McGill University). The following secondary antibodies were used: donkey anti-rabbit IgG conjugated HRP, donkey anti-mouse IgG conjugated HRP, donkey anti-rabbit IgG conjugated Texas red, donkey anti-mouse IgG conjugated Texas red (Jackson Immuno Research), protein G conjugated HRP (Molecular Probes), and sheep anti-digoxigenin conjugated FITC (Boehringer Mannheim).

### Western blots

6 × 10^5 ^293T cells were transfected as follows: 0.5 μg GagRRE, 0.1 μg SV-Hygro or SV-H6Rev, and 2.0 μg pcDNA3.1 or Sam68ΔC. For Rev-independent expression cells were transfected with 2.0 μg GagCTE, and 2.0 μg pcDNA3.1 or Sam68ΔC. Cells were harvested in whole cell lysis buffer (150 mM NaCl, 10 mM Na_2_HPO_4_, 1% Triton X-100, 0.1% SDS, 0.2% sodium azide, 0.5% sodium deoxycholate, 1 mM sodium orthovanadate), fractionated on SDS-PAGE gels and transferred to PVDF membrane (Pall Life Sciences). Bound antibodies were detected using Western Lightning (Perkin Elmer). For quantitation of western blots, films were scanned and analyzed using Imagequant software. Signals for both p55 and p24 were combined for analysis and data normalized using the tubulin signals. Quantitation shown was generated from three independent experiments.

### *In Situ *hybridization and immunofluorescence

3 × 10^5 ^HeLa cells were transfected with 0.25 μg SV H6Rev, 1.25 μg pgTat and 5.0 μg pcDNA3.1 or Sam68ΔC. 48 hours post-transfection, cells were treated with fresh media containing no drug, colcemid (0.1 μg/mL), nocodazole (1.0 μg/mL), cytochalasin D (0.5 μg/mL), or latrunculin B (0.5 μg/mL) for 2 hours prior to fixation. *In situ *hybridization was performed as previously described [56]. Digoxigenin-labeled Env-HindIII probe, antisense to HIV-1 env mRNA, was made with 10 × digoxigenin RNA labeling kit (Roche). Myc-tagged proteins were detected with monoclonal anti-myc antibody (Invitrogen) as previously described [58]. Immunofluorescence was detected using a Leica DMR microscope at either 400× or 630× magnification.

### ^35^S labeling

6 × 10^5 ^HeLa cells were transfected with 0.2 μg SV-H6Rev, 1.0 μg Gag-RRE and 4.0 μg pcDNA3.1 or Sam68ΔC. 48 hours post-transfection, cells were treated with drugs for 2 hours, as outlined above. Cells were then labeled with 100 μCi of ^35^S- methionine for 4 hours. Cells were harvested in whole cell lysis buffer then diluted with 3 volumes IPP150 (150 mM KCl, 10 mM Tris-HCl pH 7.5, 0.1% NP40, 0.1% sodium azide). The precleared lysates were incubated with anti-p24 antibody for 1 hour at 4°C, then immunoprecipitated with Gammabind plus sepharose (GE Healthcare) for 1 hour. Beads were washed with IPP150 buffer and the immunoprecipitated protein was run out on 10% SDS-PAGE. Labeled proteins were detected following exposure to a Phosphor Imager screen.

### RNA analysis

3 × 10^6 ^293T cells were transfected with 0.4 μg SV-Hygro or SV-H6Rev, 2.0 μg pgTat and 8.0 μg of pcDNA3.1, Sam68ΔC or mutants thereof. 48 hours post-transfection 25% of the cells were harvested in whole cell lysis buffer for protein analysis, and 75% of the cells were harvested for RNA isolation [59]. RNase protection assays (RPA) were performed as previously described [11]. To monitor the polyadenylation status of the pgTat mRNA 10 μg total RNA was selected using oligo(dT)25 beads according to manufacturers directions (Dynal Biotech). The selected RNA was then input into the RNase protection assay using Bl-Tat X/X probe [11]. RACE-PAT (random amplification of cDNA ends-polyadenylation test) cDNA was synthesized using an anchor primer (5'-CTCGCCGGACACGCTGAACTTTTTTTTTTTTTTTTTTTT-3') with MMLV-RT (Invitrogen). The cDNA generated was then used to generate both spliced and unspliced amplicons using the forward spliced (5'-AGCGGAGACAGCGACGAAGAG-3') or unspliced (5'-CGACCTGGATGGAGTGGGACA-3') and the reverse primer (5'-CTCGCCGGACACGCTGAAC-3'). Amplification used the following cycle parameters: 94°C, 1 minute; 66°C, 1 minute; 72°C, 2 minute; for 30 cycles. Amplicons were fractionated on native PAGE gels and detected by exposure to Phosphor Imager screen. For proviral RNA analysis, 3 × 10^6 ^293T cells were transfected with 2.0 μg HxBruR^-^/RI^- ^and 8.0 μg of Sam68ΔC or mutants thereof. The probes used for RNase protection assay were Bl-actin and Bl-SD-Gag.

Polysome analysis was performed as described by Li et al. [60] with the only modification being the addition of cytochalasin D (0.5 μg/ml) to the media 1 h prior to harvest. 0.5 ml fractions were collected following centrifugation and analyzed for protein or RNA distribution. For protein analysis, 5 μg/ml BSA was added to 200 μl of each fraction and proteins precipitated by addition of an equal volume of 40% TCA. After incubation at 4 C for 1 h, precipitate was collected by centrifugation, washed 5 times with acetone and resuspended in whole cell lysis buffer. Proteins were fractionated on SDS-PAGE gels and blotted onto PVDF membranes for detection of indicated proteins. In the case of RNA, following proteinase K/phenol-chloroform purification, RNA was ethanol precipitated, resuspended and treated with Turbo DNase 1 as per manufacturer's instructions (Ambion). RNA was then used for quantition of actin and *env *RNA by real time QRT-PCR using the following primer sets: actin forward 5'-GAG CGG TTC CGC TGC CCT GAG GCA CTC-3', actin reverse 5'-GGG CAG TGA TCT CCT TCT GCA TCC TG-3', env forward 5'-CAA CAA TGG GTC CGA GAT CTT-3', env reverse 5'-AGC TCC TAT TCC CAC TGC TC-3'. QPCR reactions were run in duplicate for each probe and gradient fraction.

### RNP immunoprecipitation

3 × 10^6 ^293T cells were transfected with 0.4 μg SVH6Rev, 2.0 μg pgTat and 8.0 μg of pcDNA3.1, Sam68ΔC or mutants thereof. Whole cell lysates were generated by a modification of the protocol of Siomi et al [61]. 48 hours post-transfection, cells were washed twice with 1 × PBS and once with Buffer A (110 mM potassium acetate, 2 mM magnesium acetate, 2 mM DTT, 10 mM HEPES pH 7.5). The cell pellet was resuspended in 400 μl Buffer B (10 mM potassium acetate, 2 mM magnesium acetate, 2 mM DTT, 5 mM HEPES pH 7.5, 20 μM cytochalasin D) and incubated on ice for 10 min. Cells were disrupted by passage through 25 gauge needle, 5 times. KCl was added to 100 mM final concentration. Cells debris was pelleted by centrifugation at 1,500 rpm. Supernatant was adjusted to 0.5% NP40 and incubated on ice for 20 min. Insoluble material was pelleted by centrifugation at 14,000 rpm and supernatant retained for use in subsequent immunoprecipitations. 1/42 of the supernatant was retained for analysis of the RNA input and 1/42 for analysis of protein input. For each immunoprecipitation, 10/42 of the soluble fraction was diluted with 10 volumes RIPA buffer (150 mM NaCl, 50 mM Tris-HCl pH 7.5, 1% NP40, 1 mM EDTA ph 7.5, 0.5% sodium deoxycholate, 0.05% SDS) and 100 u RNaseOUT (Invitrogen). The sample was precleared by incubation with Gammabind plus sepharose (GE Healthcare) for 1 h at 4°C. Supernatant was incubated overnight at 4°C with the indicated antibody. Immune complexes were collected by addition of Gammabind plus sepharose and incubation at 4°C for 1 h. The resin was washed 5× with 1 ml of RIPA buffer and then resuspended in 100 μl of RIPA. RNA was extracted from 150 μl of the resin slurry [59] and 50 μl of slurry was used for protein analysis following addition of 50 μl 2× dissociation buffer. Isolated RNA was subsequently used in RPA assays as outlined previously.

### Statistical analysis

The data are presented as mean +/- one standard deviation. Data were compared using Student's t-test. * indicates a p-value < 0.05. ** indicates a p-value < 0.01.

## Competing interests

The authors declare that they have no competing interests.

## Authors' contributions

KM performed the experiments and prepared Figures 1, 2, 3, 4, 5, 6, 7, 8, 9. VS initiated the study of Sam68ΔC. KM and AC wrote and prepared the manuscript.
